# Supplementary material for: The causal relationship between the human gut microbiota and pyogenic arthritis: a Mendelian randomization study
Source: Front Cell Infect Microbiol. 2024 Nov 26;14:1452480. doi: 10.3389/fcimb.2024.1452480 (PMC11629706; doi:10.3389/fcimb.2024.1452480)
Supplement: Supplementary file 1 [file DataSheet1.zip › mendelian test/ebi-a-GCST90027770.csv_leave_one_out.pdf]

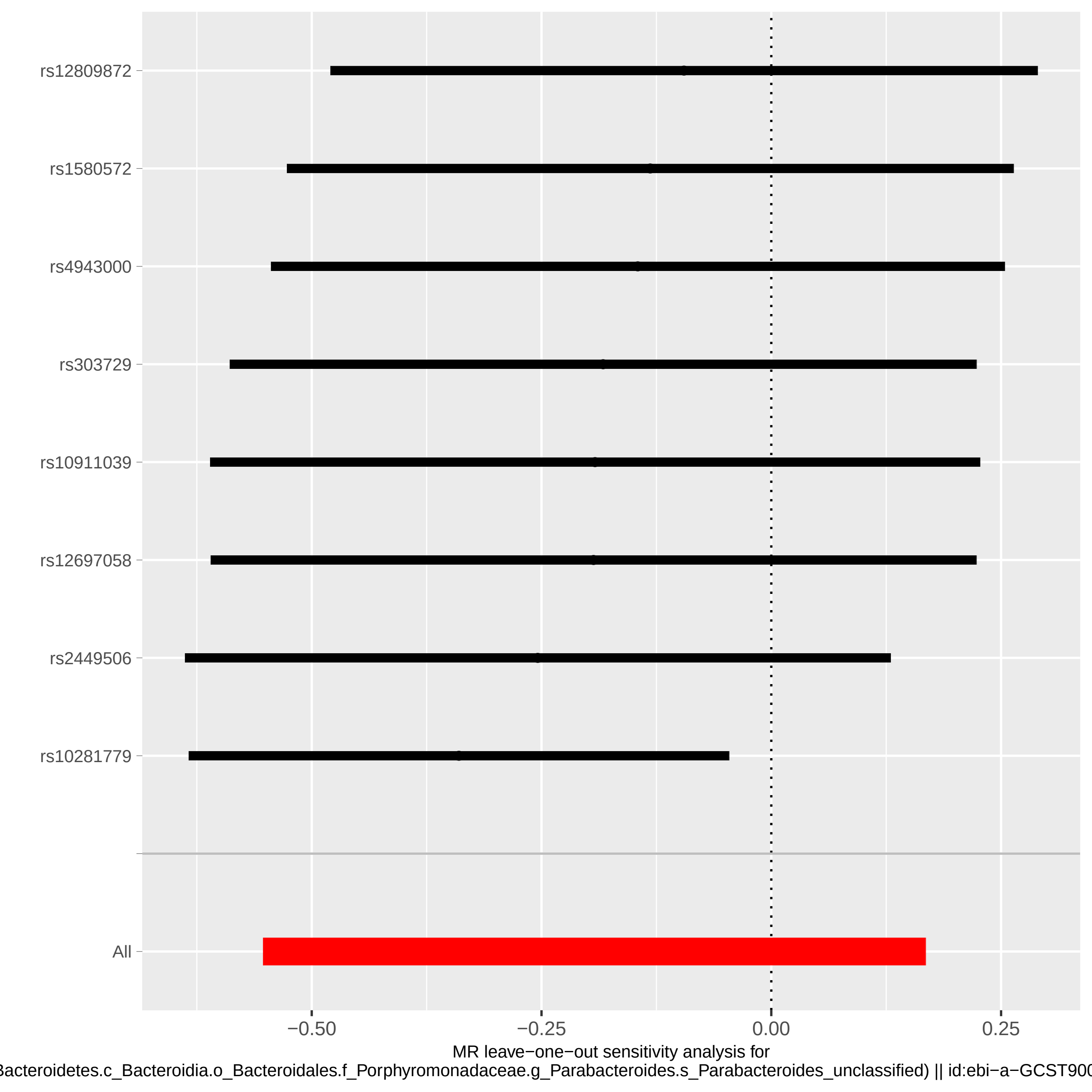

rs12809872
rs1580572
rs4943000
rs303729
rs10911039
rs12697058
rs2449506
rs10281779
All
−0.50
−0.25
0.00
0.25
MR leave−one−out sensitivity analysis for
Bacteroidetes.c_Bacteroidia.o_Bacteroidales.f_Porphyromonadaceae.g_Parabacteroides.s_Parabacteroides_unclassified) || id:ebi−a−GCST900
